# A Transgenic Flock House Virus Replicon Reveals an RNAi Independent Antiviral Mechanism Acting in *Drosophila* Follicular Somatic Cells

**DOI:** 10.1534/g3.118.200872

**Published:** 2018-12-12

**Authors:** Nelson Martins, Aurélie Lemoine, Estelle Santiago, Simona Paro, Jean-Luc Imler, Carine Meignin

**Affiliations:** Université de Strasbourg, CNRS, Insect Models of Innate Immunity (M3i, UPR9022), 67084 Strasbourg Cedex

**Keywords:** *Drosophila melanogaster*, Viral replicon, Follicular somatic cells, Antiviral Immunity, Genetics of Immunity

## Abstract

The small interfering RNA (siRNA) pathway is the main and best studied invertebrate antiviral response. Other poorly characterized protein based antiviral mechanisms also contribute to the control of viral replication in insects. In addition, it remains unclear whether tissue specific factors contribute to RNA and protein-based antiviral immunity mechanisms. *In vivo* screens to identify such factors are challenging and time consuming. In addition, the scored phenotype is usually limited to survival and/or viral load. Transgenic viral replicons are valuable tools to overcome these limitations and screen for novel antiviral factors. Here we describe transgenic *Drosophila melanogaster* lines encoding a Flock House Virus-derived replicon (FHV∆B2eGFP), expressing GFP as a reporter of viral replication. This replicon is efficiently controlled by the siRNA pathway in most somatic tissues, with GFP fluorescence providing a reliable marker for the activity of antiviral RNAi. Interestingly, in follicular somatic cells (FSC) of ovaries, this replicon is still partially repressed in an siRNA independent manner. We did not detect replicon derived Piwi-interacting RNAs in FSCs and identified 31 differentially expressed genes between restrictive and permissive FSCs. Altogether, our results uncovered a yet unidentified RNAi-independent mechanism controlling FHV replication in FSCs of ovaries and validate the FHV∆B2eGFP replicon as a tool to screen for novel tissue specific antiviral mechanisms.

Viral infections lead every year to significant losses of human lives, livestock and plant crops, and are responsible for life-threatening pandemics and emerging epidemics, such as influenza, HIV, Ebola or MERS (Middle East Respiratory Syndrome). Viruses are obligatory intracellular pathogens, with a simple RNA- or DNA-based genome sheltered in a small protein capsid, enveloped or not by cellular lipids. They hijack the cellular machinery for all stages of their life-cycle.

As in other invertebrates and plants, the main antiviral defense in the model organism *Drosophila melanogaster* is RNA interference (RNAi) (Reviewed in Ding 2010). From the three major RNAi pathways in Drosophila – microRNA, small interfering RNA (siRNA) and Piwi-interacting RNA (piRNA) – only the siRNA pathway has a well described antiviral effect. The core of the siRNA pathway comprises the Dicer2, R2D2 and Argonaute2 (Ago2) proteins. Dicer2 recognizes viral double stranded RNA (dsRNA), processing these into 21-nucleotides (nt) long, virus derived siRNAs (vsiRNA). Dicer2 bound *vs.*iRNAs, complexed with R2D2, are then transferred to the RISC complex, a large protein/RNA complex. Argonaute2 is the catalytic core of the RISC and is responsible for the degradation (slicing) of the viral genetic material, upon perfect matching of the *vs.*iRNAs with the viral genome or viral transcripts (reviewed in Paro *et al.* 2015). To counteract the RNAi pathway, several invertebrate and plant viruses evolved viral suppressors of RNAi (VSR), viral proteins that either inhibit enzymes from the siRNA pathway, such as Cricket Paralysis Virus (CrPV) 1A, or that shield viral RNAs from processing by Dicer2, such as Flock House Virus (FHV) B2 or Drosophila C Virus (DCV) 1A (reviewed in Bronkhorst and van Rij 2014; Gammon and Mello 2015).

The piRNA pathway has also been proposed to participate in antiviral immunity in insects. This pathway is mainly active in the reproductive tissues, protecting the genome against transposable element (TE) mobilization (Reviewed in Malone and Hannon 2009). piRNAs, which have distinct biogenesis and sequence features than those of siRNAs (24-30 nt, with strand-specific nucleotide biases), can be generated either from active transposons or from specific genomic loci (piRNA clusters) (Brennecke *et al.* 2007). piRNA clusters, composed mostly of truncated versions of TEs, are thought to serve as a memory of TE invasions (Yamanaka *et al.* 2014). Virus derived piRNAs have been observed in some Drosophila cell lines (Wu *et al.* 2010) and some arthropod species (Morazzani *et al.* 2012; Vodovar *et al.* 2012; Schnettler *et al.* 2013; Léger *et al.* 2013; Lewis *et al.* 2018), revealing that the piRNA pathway can be activated by viruses. However, an antiviral function for this pathway could not be established in *D. melanogaster* (Petit *et al.* 2016).

In addition to RNAi, other pathways also participate in restricting viral infections in invertebrates. The major innate immunity pathways in insects, Toll and Imd, contribute to antiviral resistance (Costa *et al.* 2009; Avadhanula *et al.* 2009; Ferreira *et al.* 2014; Lamiable *et al.* 2016b). The STING and Jak/Stat pathways, part of the interferon response in vertebrates, are also triggered upon viral infection in Drosophila (Dostert *et al.* 2005; Merkling *et al.* 2015; Goto *et al.* 2018). Of note some genes are induced by Dicer2, via an as yet unknown signal transduction pathway, connecting RNAi and inducible responses (Deddouche *et al.* 2008). Induced antiviral immunity is largely virus specific, with few genes (*e.g.*, heat shock proteins, *diedel*, *vago*, *vir-1*, *nazo*) being upregulated upon infection by more than one virus. Other cell stress and signaling pathways, such as autophagy or ubiquitination, control viruses, acting either as restriction or pro-viral factors (Shelly *et al.* 2009; Martins *et al.* 2014; Merkling *et al.* 2015; Nainu *et al.* 2015; Lamiable *et al.* 2016a). However, their role and relative importance in complementing the RNAi response is not completely characterized (reviewed in Mussabekova *et al.* 2017).

*D. melanogaster* is ideally suited for unbiased large-scale genetic screens (St Johnston 2002), which could help to identify new antiviral genes. In the context of infections, however, one limitation of such screens is the need to inject a standardized inoculum for the experimental infection, which can be cumbersome and time consuming. Use of transgenic viral replicons can bypass this bottleneck (Lu *et al.* 2009; Avadhanula *et al.* 2009; Wernet *et al.* 2014; Coffman *et al.* 2017).Originally isolated from the grass grub *Costelytra zealandica* (White) (*Coleoptera: Scarabaeidae*), the alphanodavirus FHV is a model insect virus that has been used to decipher antiviral immunity in Drosophila (Li *et al.* 2002; Galiana-Arnoux *et al.* 2006; Eleftherianos *et al.* 2011; Kemp *et al.* 2013). It has a bipartite positive sense ssRNA genome comprising RNA1 (3.1kb) and RNA2 (1.4kb). RNA1 encodes the RNA-dependent RNA polymerase and contains a frame-shifted subgenomic RNA 3 (369 nt) that encodes the VSR B2, a dsRNA binding protein (Lu *et al.* 2005; Han *et al.* 2011; Petrillo *et al.* 2013). RNA2 encodes the capsid protein. RNA1 is sufficient for autonomous replication in insect cells (Johnson and Ball 1999), but also in nematodes, yeast and plants (Li *et al.* 2002). The FR1∆B2GFP replicon (hereafter FHV∆B2eGFP) was initially developed by Ding and colleagues to characterize antiviral immunity in *D. melanogaster* cells and in the nematode *Caenorhabditis elegans* (Li *et al.* 2004; Lu *et al.* 2005, 2009; Wang *et al.* 2006). It is a transgenic form of the RNA1-segment of the FHV genome, where the majority of the coding sequence for B2 was replaced by eGFP. FHV∆B2eGFP is efficiently silenced by the siRNA pathway in wild-type cells, such that GFP expression is not detectable. However, when co-expressed with B2 or other VSRs, or in a siRNA pathway mutant context (Lu *et al.* 2005, 2009; Wang *et al.* 2006), it faithfully recapitulates the replication steps of a viral infection, with robust GFP expression.

Here, we describe transgenic lines of *D. melanogaster* expressing FHV∆B2eGFP, under the control of the Gal4/UAS system. We show that the replicon is efficiently controlled by the siRNA pathway in most somatic tissues, suggesting that it can be used to identify novel cofactors of the known core components of the siRNA pathway. Interestingly, this replicon also allowed us to uncover an siRNA independent mechanism that maintains the replicon silent in most follicular somatic cells of ovaries, suggesting the existence of an independent layer of defense against viral replication in this tissue.

## Material and Methods

### Drosophila strains and culture

The used Drosophila strains are described in Table I of File S1. Flies were kept on standard cornmeal–agar medium at 25°. All fly lines were tested for Wolbachia infection and cured whenever necessary (Teixeira *et al.* 2008).

### Cloning of FHV∆B2eGFP and transgenesis

The UAS-FHV∆B2eGFP transgene was constructed by using a full-length FHV replicon developed by Ding and colleagues (Li *et al.* 2004). The FHV∆B2eGFP replicon was subcloned in pBluescriptII KS(+). A *Not*I-*Kpn*I fragment containing the FHV∆B2eGFP replicon was cloned downstream of the promoter in the pUASt transformation vector. Two independent transgenic lines were obtained in w^1118^ background. Vector maps and sequences of the transgene are provided in File S1.

### Small RNA sequencing

Ovaries (20 pairs) from 3-4 days old flies were dissected, collected into Trizol (Thermo Fisher Scientific,Waltham, MA, USA), snap frozen in dry ice and kept at -80° until subsequent RNA preparation. RNA extraction from Trizol stored ovaries was done following manufacturers’ instructions. Quality of extracted RNA was assessed in Agilent Bioanalyzer 2100 (Agilent, Santa Clara, CA, USA), using the Eukaryote Total RNA Nano assay.

Libraries for small RNA sequencing were prepared using the Illumina TruSeq Small RNA library preparation kit (Illumina, San Diego, CA, USA), according to manufacturers’ protocol, with the following modifications: after ligation of the 3′ adapter, 20 pmol of a terminator oligonucleotide was added to deplete the libraries from 2S ribosomal RNA (rRNA)(Wickersheim and Blumenstiel 2013), samples were reheated for 2 min at 70° and placed on ice, after which the protocol was resumed.

Library quality was assessed in Agilent Bioanalyzer 2100 (Agilent), using the High Sensitivity DNA assay. Libraries were sequenced in Illumina HiSeq 4000 (Illumina), with a single-end 50 bp read-length strategy. Sequencing was performed by the IGBMC Microarray and Sequencing platform, a member of the ‘France Génomique’ consortium (ANR-10-INBS-0009).

After quality trimming and adapter removal using Trimmomatic v0.36 (Bolger *et al.* 2014), reads were mapped sequentially using bowtie v1.1.2 (Langmead *et al.* 2009) to the following references: rRNA’s, microRNA’s, FHV∆B2eGFP, TEs and Drosophila genome (dm6), using piPipes v1.5.0 (Han *et al.* 2015), allowing 1 mismatch for all references except for the Drosophila genome (2 mismatches allowed). Library and mapping statististics are shown in Table S1. Analysis of mapped reads was done using custom R scripts. Counts of reads mapping to FHV∆B2eGFP and *gypsy* were normalized against the total number of reads excluding ribosomal RNA and micro RNA mapping reads. Other normalization strategies yielded qualitatively identical results.

### Follicle somatic cells isolation and cell sorting

Dissociation of follicle somatic cells were done according to Bryant *et al.* (1999) with the following modifications: ovaries (40-60 pairs) from 3-4 day old flies were dissected in PBS; cells were dissociated in Cell Dissociation Solution (Sigma-Aldrich, St. Quentin Fallaviers, France) with 50 ug/ml liberase DL (Sigma-Aldrich) and resuspended in OSS cell medium (DGRC, https://dgrc.bio.indiana.edu/product/View?product=190) without added fly extract. FACS analysis and cell sorting was done on the FACS Aria II or FACS Aria Fusion (BD Biosciences, San Jose, CA, USA), configured to detect DAPI, GFP and mCherry according to the manufacturers’ instructions. Alive cells, not incorporating DAPI, were sorted into mCherry^-^/GFP^-^, mCherry^+^/GFP^-^ and mCherry^+^/GFP^+^ populations in 0.5 ml of OSS cell medium. Gating was done in real time using FACS Diva (v8.0.1, BD Biosciences). FACS sorting was done at the Flow Cytometry platform at Institut de Génétique et de Biologie Moléculaire et Cellulaire (IGBMC, Strasbourg, France). Cells were centrifuged at 1000 X g for 5 min, resuspended in 0.4 ml of Trizol, snap frozen in dry ice and kept at -80° before subsequent RNA preparation.

### Long RNA sequencing and bioinformatic analysis

RNA extraction from Trizol stored cells was done following manufacturers’ instructions. Libraries for total RNA sequencing were then prepared using the NUGEN Ovation Drosophila RNA-seq system (NuGEN, Leek, The Netherlands), according to the manufacturers’ instructions. cDNA was fragmented using a Covaris S-series System (Covaris, Brighton, UK), to obtain a median fragment size of 300-bp. Library quality was assessed in Agilent Bioanalyzer 2100 (Agilent), using the High Sensitivity DNA assay. Libraries were sequenced in Illumina HiSeq 4000 (Illumina, San Diego, CA, USA), with a single-end 50 base-pairs (bp) read-length strategy. Sequencing was performed by the IGBMC Microarray and Sequencing platform.

After quality trimming and adapter removal using Trimmomatic, reads were mapped using STAR v2.5.2b (Dobin *et al.* 2013) to a modified Drosophila genome and annotation (r6.04 with the sequences/annotations of the FHV∆B2eGFP, UAS-CrPV-1A and UAS-mCherry∷NLS transgenes the transgenes added as extra chromosomes). Reads mapping to the sense strand of the transcripts or transgenes were counted with featureCounts v1.5.2 (Liao *et al.* 2014) using the modified Drosophila annotation files. Differential gene expression of transcripts present in ≥25% of the libraries with at least 5 reads across all libraries was done using DESeq2 v1.20 (Love *et al.* 2014). Variance was estimated using the local fitting method. Read counts and normalized read counts are shown in Tables S4-S5. Transcripts with log2 difference in expression ≥ 1.5 and Benjamini & Hochberg corrected *p*-value < 0.05 were considered differentially expressed.

### Fixation of ovarian tissue

Ovary staining and visualization was carried out according to published protocols. Briefly, ovaries of 3-4 day old flies were dissected in PBTX (PBS+0.1% Triton X), fixed for 20-25 min in a 1:4 solution of 1% methanol-free formaldehyde (ThermoFisher Scientific) in 1X PBTX:Heptane, after dissociation by pipetting up and down with a 1000 ul pipette, and washed three times in PBTX for 5 min.

### Immunohistochemistry

For immunostaining, samples were blocked for 30 min in PBTX+1% BSA, and primary antibodies, diluted in PBTX+1% BSA were incubated overnight at 4°. After washing 3 times with PBTX+0.1% BSA, secondary antibodies, diluted in PBTX+0.1% BSA were incubated for one hour at room temperature. Antibodies and dilutions used in this study are mouse anti-GFP (Roche #11814460001, 1:1000), rabbit anti-FHV-RdRP (gift from A. Schneeman, 1:500), mouse monoclonal anti-dsRNA (J2; English & Scientific Consulting, Szirak, Hungary, 1:500). After washing 3 times with PBTX+0.1% BSA, secondary antibodies from Invitrogen were goat anti-rabbit or anti-mouse with Alexa-564 and Alexa-594. Stained ovaries were mounted in Vectashield with DAPI (Vector Laboratories, Peterborough, UK) on glass slides.

### Fluorescent in-situ hybridization

For fluorescent *in-situ* hybridization, Custom Stellaris FISH Probes were designed against the first 2600 nucleotides of the sense strand of FHV-RNA1 by utilizing the Stellaris RNA FISH Probe Designer (Biosearch Technologies, Inc., Petaluma, CA) available online at www.biosearchtech.com/stellarisdesigner (version 4.2). The samples were hybridized with the FISH Probe set labeled with Quasar 640 (Biosearch Technologies, Inc.), adapting the manufacturer’s instruction for hybridization of Drosophila imaginal discs (available online at www.biosearchtech.com/stellarisprotocols) to a tube format. Briefly, fixed ovaries were incubated with Stellaris Wash Buffer A for 5 min, and hybridized with probes (125 nM) in Stellaris Hybridization Buffer in the dark at 37° overnight. After washing twice with Wash Buffer A at 37° in the dark for 30 min and once with Stellaris Wash Buffer B for 5 min at room temperature, samples were mounted in Vectashield with DAPI on glass slides.

### Imaging

Confocal images were taken on a Leica LSM700 or 780 confocal microscope; widefield and stereromicroscope images were taken on a Zeiss Axiovert 200M and a Zeiss Stereo Discovery V12, respectively. Images were processed with ImageJ software.

### Data Availability

Strains and reagents (described in File S1) and analysis scripts are available upon request. Supplementary figures S1-5, tables S1-5 and File S1 are available at figshare. Raw sequencing data are available from the European Nucleotide Archives with references PRJEB28601 (small RNA sequencing, relative to Figures 4 and S2-4 and tables S1-2) and PRJEB28602 (RNA sequencing of sorted follicular somatic cells, relative to Figures 5 and S5 and tables S3-5). Supplemental material available at Figshare: https://doi.org/10.25387/g3.7423376.

## Results

### A transgenic replicon to monitor antiviral RNAi in vivo

**Figure 1:**
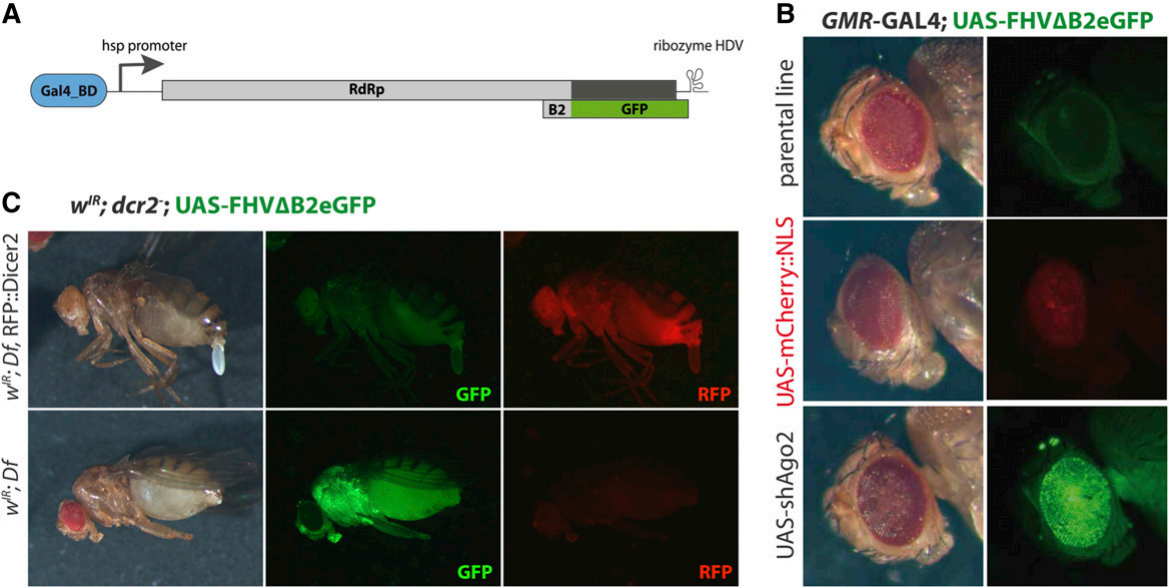
The siRNA pathway is required to control FHV∆B2eGFP replication and GFP expression in *Drosophila melanogaster* somatic tissues. (A) Schematics of the FHV∆B2eGFP replicon transgenic construct. The CDS for the viral suppressor of RNAi B2 in the FHV genome was replaced by GFP except for the initial 23 codons. (B) *GMR*-GAL4 driven expression of FHV∆B2eGFP in eyes. The replicon is co-expressed with UAS-mCherry::NLS or in RNAi deficient background (UAS-shAgo2). (C) In *dicer2* null mutant background, the expression of FHV∆B2eGFP is detected in adult stages. The ubiquitous expression of RFP::Dicer2 controls the expression of FHV∆B2eGFP.

The FHV∆B2eGFP replicon was placed under the control of a minimal heat-shock promoter and yeast UAS_Gal4_ sequences (Figure 1A and File S1), and the resulting construct was injected in embryos. Only two independent transgenic lines were obtained, probably reflecting the detrimental effects of the ectopic leaky expression of an RNA-dependent RNA polymerase (RdRP). Indeed, crossing the replicon lines with strong ubiquitous driver lines (*e.g.*, *actin*-Gal4) did not yield viable progeny. The use of tissue specific drivers allowed to test the control of the replicon in different target tissues.

Expression of the replicon in eyes using the *GMR*-Gal4 driver did not produce detectable GFP fluorescence, even though the driver efficiently drove expression of a UAS-mCherry∷NLS reporter in this tissue (Figure 1B). When the flies also expressed a small hairpin (sh) RNA targeting Ago2 (*GMR*-Gal4; UAS-FHV∆B2eGFP/UAS-shAgo2), a strong and homogeneous green fluorescence was observed in the eyes (Figure 1B). Co-expression of the replicon and the VSR CrPV-1A or FHV-B2, or with different drivers active in other somatic tissues also resulted in derepression of GFP expression (not shown). In RNAi mutant flies (*e.g.*, *dcr2*), we observed GFP signal in the whole body in the absence of driver, reflecting baseline leaky expression amplified by the RdRP (Figure 1C) (Deddouche *et al.* 2008). These results indicate that the UAS-FHV∆B2eGFP replicon can be used to monitor the activity of antiviral RNA interference *in vivo*.

### The FHV∆B2eGFP replicon is not exclusively controlled by the siRNA pathway in follicular somatic cells of the ovary

**Figure 2:**
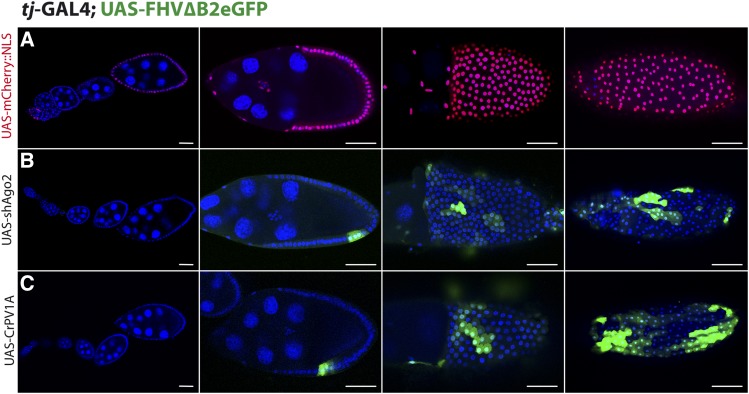
The siRNA pathway is not required for partial control of FHV∆B2eGFP in follicular somatic cells of the Drosophila ovary. *tj*-GAL4 driven expression of FHV∆B2eGFP in follicular somatic cells during oogenesis. The replicon is co-expressed with UAS-mCherry::NLS (A) or in RNAi deficient background (UAS-shAgo2 (B) or UAS-CrPV-1A (C)). Early to late stages of oogenesis are represented. Scale bar - 25µm.

**Figure 3:**
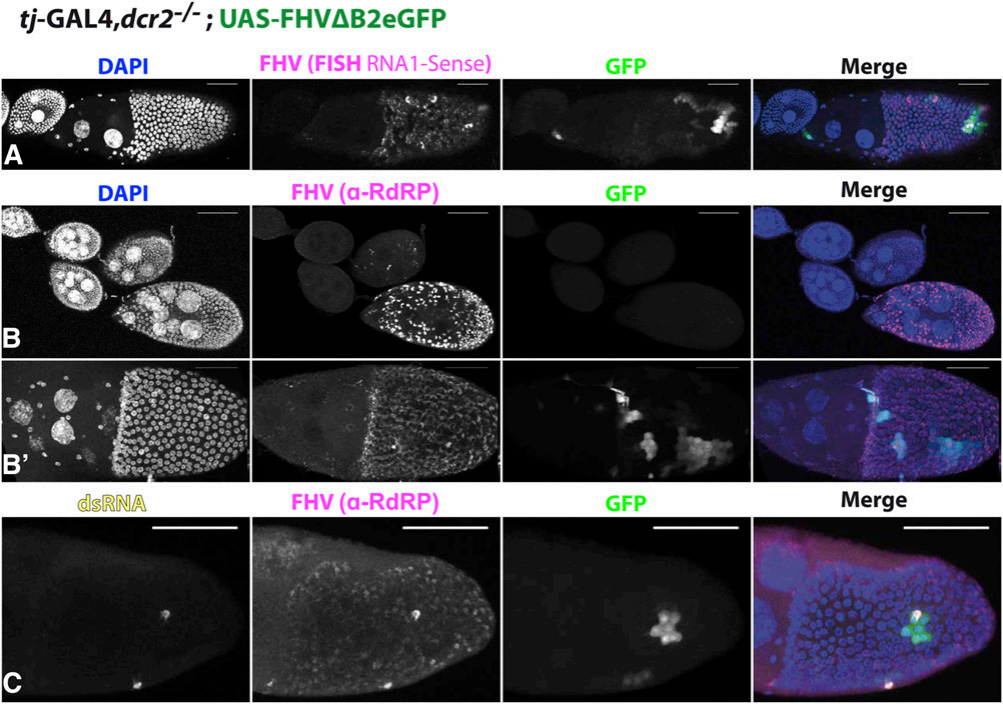
FHV∆B2eGFP is homogeneously expressed but active replication is restricted to discrete foci. *tj*-GAL4 driven expression of FHV∆B2eGFP in follicular somatic cells during oogenesis. (A) Fluorescent *in-situ* hybridization (FISH) of the RNA1 of FHV; (B,B’) Immunostaining for the RdRP of FHV and (C) for the RdRP and for double stranded RNA dsRNA (J2-antibody). Scale bar - 50µm.

Driving the expression of the replicon with germline specific drivers (*e.g.*, *mat.αTub67c*-Gal4) led to sterile progeny with rudimentary ovaries (not shown). When testing the replicon with a follicular somatic cell (FSC) specific driver, in a siRNA impaired background (*tj*-Gal4; UAS-FHV∆B2eGFP/UAS-shAgo2), we observed only a partial derepression of GFP expression. Fluorescence was consistently observed in restricted patches (Figure 2B) even though the driver efficiently and homogeneously drove expression of a UAS-mCherry∷NLS reporter in this tissue (Figure 2A and S1A). Moreover, while mCherry expression could be seen in early stages of oogenesis (stage 5-6), replicon-derived GFP expression was only observed after stage 8 of oogenesis, with GFP patches becoming more apparent after stage 10. Generally, GFP was detected in the main body cells, which comprise the majority of the follicular somatic cells, in a non-stereotypical pattern. We also could also detect GFP in the stretched cells that overlie the nurse cells (*e.g.*, figure 3A), and in the posterior (Figure 2B) or centripetally migrating cells (*e.g.*, figure 3B’). Co-expression of the replicon and VSRs or expression of the replicon in *dcr2* or *Ago2* mutant flies gave similar results (Figure 2C and Figure S1A-B).

Variegated GFP expression, due to a heterogeneous activation of the transgene, was observed for several driver/reporter pairs in FSCs (Skora and Spradling 2010; Lee *et al.* 2017). When co-expressing the replicon and the VSR CrPV-1A in mutant backgrounds that dominantly suppress variegation, similar patches were observed (Figure S1C). To further rule out a variegation effect, we monitored FHV-RNA1 expression using Fluorescent *in-situ* Hybridization (FISH) and FHV-RdRP expression by immunostaining. In both cases we detected expression in all cells, ruling out a transcriptional or translational effect (Figure 3A-B,B’). Of note, a few scattered cells expressed higher levels of RNA, overlapping with GFP positive cells. One hallmark of viral replication is the accumulation of double-stranded (ds) RNAs. Interestingly, dsRNA could only be detected in single isolated cells, falling within patches of GFP positive cells (Figure 3C). This suggests that even though the siRNA pathway is inactivated, only a limited number of FSCs support active viral replication. We hypothesize that free GFP expressed in these cells spreads to neighboring cells through ring canals (McLean and Cooley 2013). In summary, our results reveal that when the siRNA pathway is impaired the replicon remains controlled in the majority of FSCs.

### The piRNA pathway is not involved in replicon control in the FSCs

**Figure 4:**
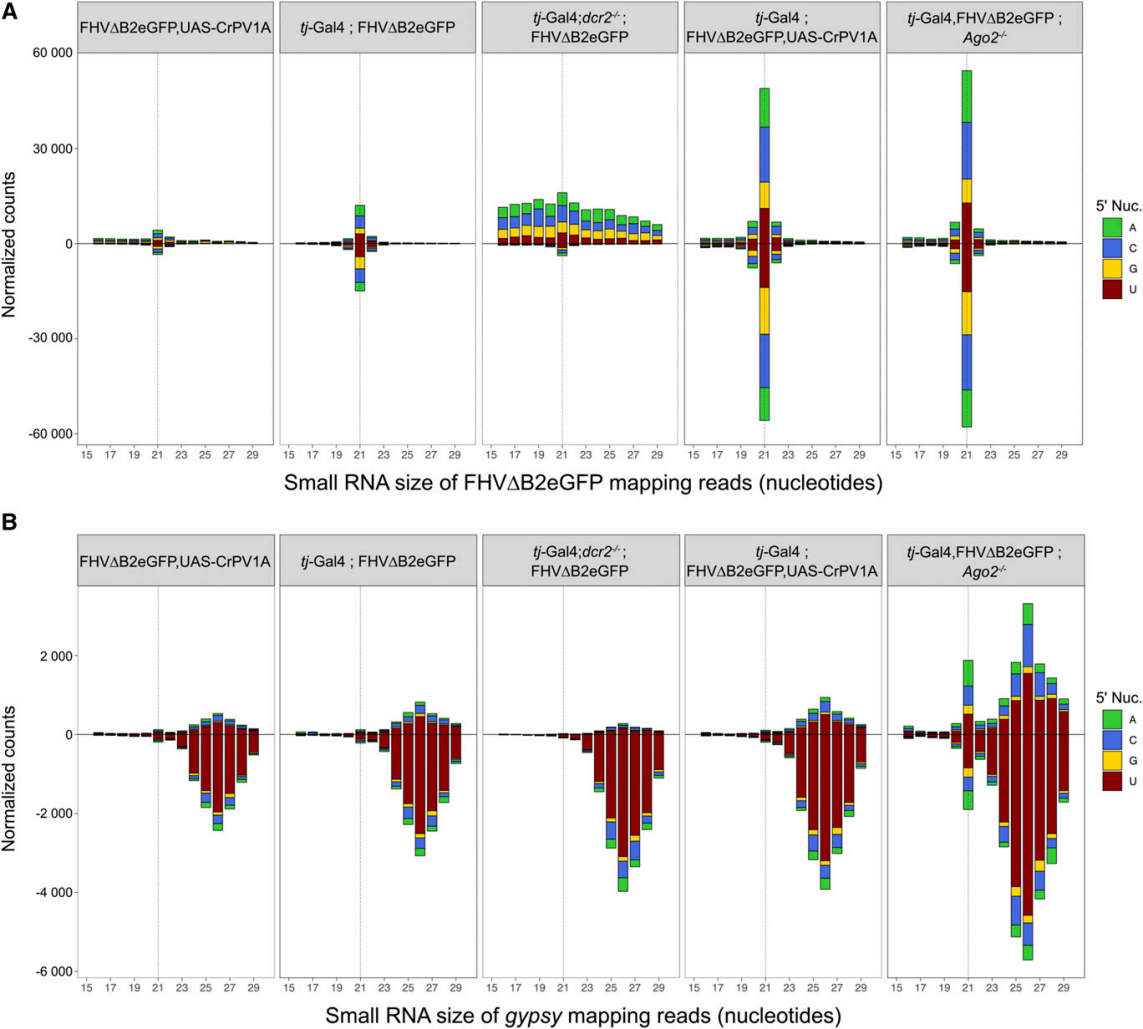
The piRNA pathway is not involved in FHV∆B2eGFP control. Size distribution and 5′ nucleotide of small RNA reads mapping to FHV∆B2eGFP (A) or to *gypsy* transposable element (B), in small RNA libraries prepared from ovaries of *Drosophila melanogaster*. Reads are normalized to total reads, excluding microRNAs and ribosomal RNAs.

The primary piRNA pathway is active in FSCs and is essential for the control of transposon replication in this tissue. To assess the involvement of this pathway in the control of the replicon, we sequenced the small RNAs produced in replicon expressing ovaries. In the absence of the driver (UAS-FHV∆B2eGFP, UAS-CrPV-1A), there was a low number of reads mapping to the replicon, indicative of baseline leaky expression (Figure 4A, left panel). Upon driving with *tj*-Gal4, a peak of 21nt small RNAs mapping in equal proportion to the (+) and (-) strands was observed. This sharp peak was not observed in libraries prepared from *dcr2* null mutant flies, confirming that it corresponds to virus-derived siRNAs (Figure 4A, S2A, middle panel). In *Ago2* null mutant flies, or expressing CrPV-1A, we noted a strong raise in the number of siRNAs, reflecting the increased replicon activity in these backgrounds (Figure 4A, right panels). Of note, other targets of the siRNA pathway (hairpin RNAs or *cis*-natural antisense transcripts) (Kawamura *et al.* 2008; Czech *et al.* 2008; Wen *et al.* 2014, 2015) showed the expected pattern of regulation in *dcr2* and *Ago2* mutant flies (Figure S3 and Table S2). In none of these genetic backgrounds however did we observe small RNAs bearing the signature for canonical piRNAs, such as 24-28nt size range or enrichment for U at the 5′ end, although piRNAs targeting TEs, such as *gypsy*, were present in the libraries (Figure 4B and Figure S2D). We consistently observed an accumulation of 21-nt long siRNAs for most transposons in *Ago2* null mutant background (Figure S4), highlighting the importance of the siRNA pathway in the processing of TEs (Ghildiyal *et al.* 2008; Kawamura *et al.* 2008; Chung *et al.* 2008; Czech *et al.* 2008). We conclude that only the siRNA pathway is activated by the replicon in FSCs and that the second layer of defense does not rely on the production of piRNAs.

### Characterization of the transcriptome of permissive and restrictive FSCs

**Figure 5:**
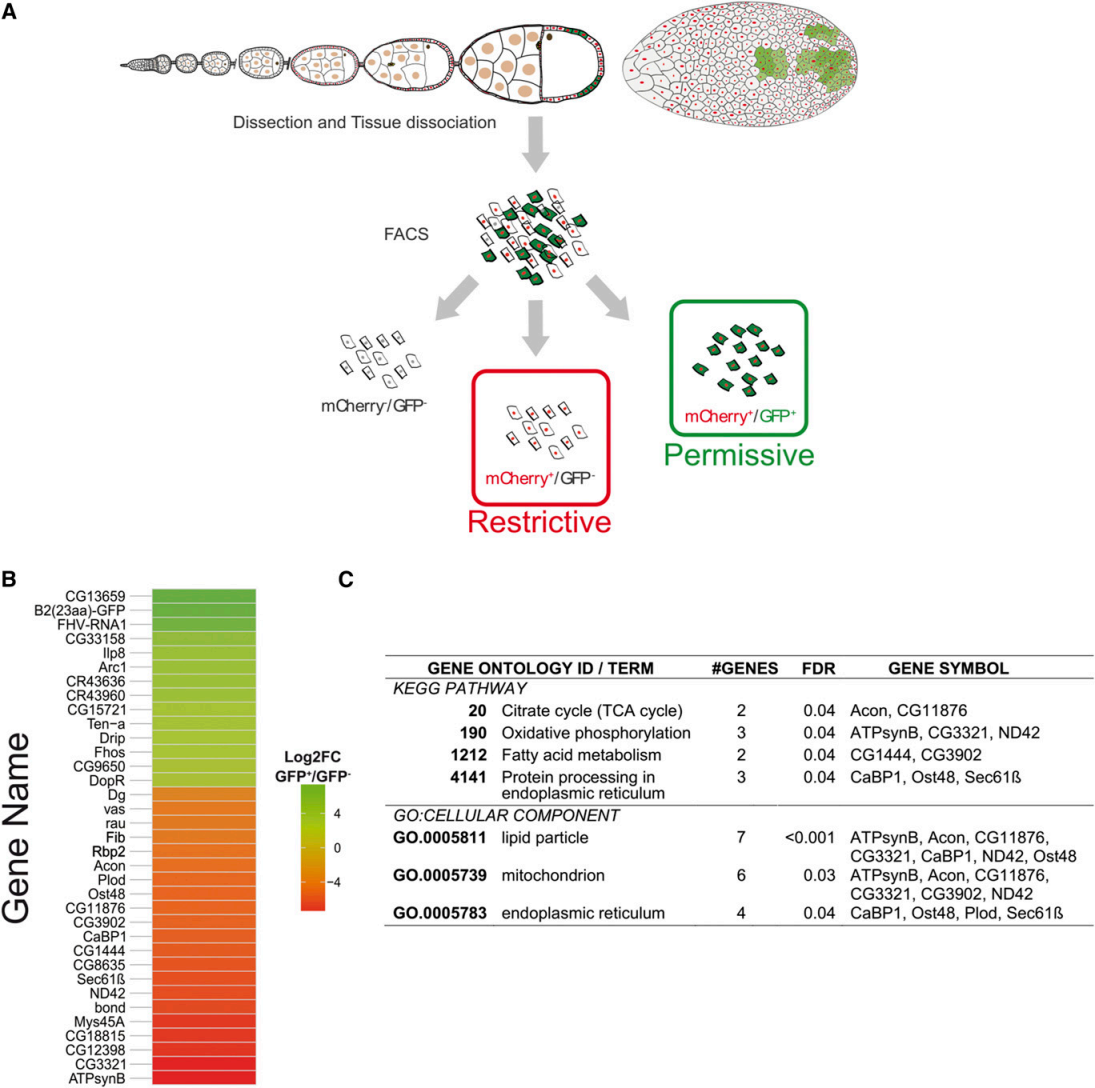
Transcriptional profiling of FHV∆B2eGFP restrictive and permissive FSCs reveals candidate differentially expressed genes. (A) Overview of the follicular somatic cells separation strategy. Total RNAseq (NuGEN) was done on sorted FSCs of *tj*-Gal4,UAS-mCherry::NLS; UAS-FHV∆B2eGFP,UAS-CrPV-1A flies. (B) Heatmap of the 35 differentially expressed genes between FHV∆B2eGFP restrictive (mCherry^+^/GFP) and permissive (mCherry^+^/GFP^+^) FSCs including two ncRNAs and two transcripts from the replicon itself. (C) Enriched gene ontology categories of the differentially expressed genes from the restrictive fraction, according to STRING v10.5 (string-db.org).

We dissected the ovaries from flies expressing the replicon, CrPV-1A and a mCherry∷NLS reporter in FSCs. After dissociating cells according to Bryant *et al.* (1999), we sorted FSCs based on mCherry expression, further separating them into in restrictive- (GFP^-^) and permissive- (GFP^+^) enriched pools (Figure 5A). Total RNAs were extracted from these pools and analyzed by RNA sequencing. We first analyzed reads mapping to the FHV∆B2eGFP replicon, in particular at the differences between RNA1 and RNA3. As expected, we observed increased levels of RNA1 in the permissive cells. Importantly, mean coverage of B2(23aa)∷eGFP was 1.ninefold higher than that of the whole RNA1, indicating independent RNA1 and RNA3 transcription, as occurs during FHV replication (Ball and Johnson 1998). In the restrictive cells, RNA1 and RNA3 had similar coverage distribution, pointing to a blockage of viral replication (Figure S5). There was a total of 35 differentially expressed (DE) transcripts between the two pools (Figure 5B, Table S3), corresponding to 31 protein coding genes, two non-coding RNAs and two transcripts from the FHV∆B2eGFP transgene. FHV-RNA1 and B2(23aa)∷eGFP were among the top DE transcripts in the permissive pool, further validating the sorting. 12 other transcripts, including two non-coding RNAs, were enriched in the FHV∆B2eGFP permissive cells. Gene ontology (GO) analysis did not reveal enrichment in any functional category. We note however that the unknown gene *CG13659*, which was enriched by 16-fold in permissive cells, is induced upon systemic acute infection by FHV (Kemp *et al.* 2013). This suggests that some of the DE transcripts could represent genes involved in a response of FSCs to viral infection or could reflect cellular damages caused by active viral replication. For instance, of 21 transcripts enriched in the restrictive fraction, 9 were associated with the mitochondrial compartment and/or fatty acid metabolism (*P* < 0.04 for all GO terms, Figure 5B-C). The decreased expression of these genes in the permissive pool could reflect alterations of mitochondrial metabolism or damage caused by replication of viral RNA in invaginations of the outer membrane (Miller *et al.* 2001; Kopek *et al.* 2007; Ertel *et al.* 2017).

## Discussion

Here, we describe transgenic lines of *D. melanogaster* expressing the viral replicon FHV∆B2eGFP, and report that the replicon is efficiently controlled by the siRNA pathway in most somatic tissues. In ovaries of siRNA pathway impaired flies, however, the replicon remained silent in the majority of follicular somatic cells. The FSCs, forming an epithelial layer that surrounds each germline cyst, are essential for oogenesis, providing the maturing egg chamber with positional cues necessary for the establishment of the anterior-posterior and dorsal-ventral polarity and giving rise to the eggshell (Wu *et al.* 2008). They are also vitellogenic, participating with the fat body in the provisioning of yolk to the egg. Moreover, they form the last epithelial barrier for pathogen transmission between the hemolymph and the germline, although not much is known about the immune function of these cells. There are few examples of how transovarially transmited parasites/symbionts are able to infect the germline of their hosts. Two bacterial species are known to be transovarially transmitted in *D. melanogaster*, *Wolbachia pipientis* and *Spiroplasma poulsonii*. Wolbachia, obligate endosymbionts, are detected both in the FSCs and in the oocyte (Toomey *et al.* 2013). On the contrary, the facultative endosymbiont Spiroplasma transits directly from the hemolymph to the oocyte bound to yolk proteins secreted by the fat body, being detected only in the extracellular space between FSCs (Herren *et al.* 2013). Interestingly, two transovarially transmitted arthropod viruses (TyCLV and Rice Stripe Virus) also bind to yolk proteins to infect the oocytes of their hosts and were never detected infecting follicle cells, despite being able to infect neighboring somatic tissues (Huo *et al.* 2014; Wei *et al.* 2017). Sigma virus is the only known transovarially transmitted virus in Drosophila. Of note, the efficiency of vertical transmission of Sigma is lower in flies injected with the virus than in persistently infected flies, even though viral titers reach higher levels (Longdon and Jiggins 2012). Taken together and although studies looking at these tissues with viruses that are not transovarially transmitted are scarce (*e.g.*, Thomson *et al.* 2012), these observations suggest that the follicular somatic cells are refractory to most infections. In agreement with this idea, our data reveals that a viral replicon can be controlled by at least two mechanisms in FSCs, the siRNA pathway and a novel restriction mechanism.

In Drosophila, one difference between FSCs and other somatic tissues is the activity of the primary piRNA pathway, which controls transposon activity in FSCs. In most somatic tissues, transposons are controlled by the endogenous siRNA pathway (endo-siRNA), whose main effector proteins are the same as the antiviral siRNA pathway (Czech *et al.* 2008). Most of the piRNAs produced in FSCs originate from piRNA clusters, such as *flamenco* (Malone *et al.* 2009). *flamenco* mutants show high transposition rates of retrotransposons, namely of *gypsy*, and follicle cells of these mutants produce viral like particles, revealing the importance of piRNAs in FSCs for the control of transposons. These particles can then pass to the germline, before the formation of the vitelline membrane (stage 10 of oogenesis) allowing *gypsy* to integrate in the genome (Pélisson *et al.* 1994; Song *et al.* 1997). Hence, the production of primary piRNAs in FSCs was proposed to be an evolutionary solution to counteract the expression of retrotransposons, which have a retroviral origin, in these cells (Malone *et al.* 2009; Li *et al.* 2009). Our hypothesis that the piRNA could play a role in the control of the replicon in this tissue was further strengthened by *i)* the observation that an FSC derived cell line (the OSS cell line), produces virus derived piRNAs from FHV and DCV (Wu *et al.* 2010) and *ii)* the proposed role of the piRNA pathway in the control of viruses (Reviewed in Miesen *et al.* 2016). However, our results show unambiguously that there are no FHV∆B2eGFP derived piRNAs produced in the ovaries of transgenic flies, even in siRNA mutants. This confirms the results of other groups, which indicate that an antiviral piRNA pathway might be restricted to a few arthropod species (Lewis *et al.* 2018), not including Drosophila (Petit *et al.* 2016).

If not RNA-based, a protein-based mechanism may be involved in the restriction of the FHV replicon. The transcriptome data of sorted FSCs reported here will be a useful resource to identify candidate genes involved in this mechanism. These candidates may reflect a response to infection and the induction of an antiviral state. For example, *CG13659* and the Drosophila insulin-like peptide 8 (*dIlp8*), are both expressed at higher levels in the permissive cell fraction. Orthologs of *CG13659* are present in most arthropods and in *D. melanogaster* it is induced upon systemic infection by FHV (Kemp *et al.* 2013). It encodes a protein of unknown function with a choline kinase like domain. dIlp8 is secreted by imaginal discs upon damage, arresting growth during Drosophila development to allow tissue repair (Colombani *et al.* 2012). It is known to be expressed in ovaries (flybase.org), but its role in this tissue has not yet been defined. Our results suggest that it could serve as a cytokine issued by permissive cells to induce restriction in neighboring cells. Of note, constitutively expressed restriction factors, regulated at the post-transcriptional level, may also control infection. In this regard, the identified DEGs could be linked to cellular alterations triggered by viral RNA replication (*e.g.*, changes in pathways associated with the mitochondrial compartment (Go *et al.* 2006; Castorena *et al.* 2010)).

Whatever the mechanism, the transgenic FHV∆B2eGFP lines we describe here provide a useful tool for unbiased genetic screens aimed at characterizing it. Such unbiased genetic screens in another model organism, the nematode *C. elegans*, recently identified a novel, RNAi-independent restriction mechanism for RNA viruses based on the 3′uridylation of viral transcripts by terminal uridyl transferases (Le Pen *et al.* 2018). The FHV∆B2eGFP transgenic lines will also be valuable tools for the characterization of other tissue specific or developmentally restricted antiviral mechanisms. Cell based knockdown and mutagenesis screens, while useful to elucidate cell-intrinsic mechanisms, fail to capture the complexity and signaling pathways present in tissues or organisms, and the responses are contingent on the ontology of the cell line. On the other hand, *in vivo* screens with injected viruses are time consuming, bypass the natural route of infection for most viruses, and the scored phenotype is usually limited to survival and/or whole-body viral load. Unbiased screens using infectious viruses targeting different tissues, routes of infection and developmental stages present even greater logistic and standardization challenges. The FHV∆B2eGFP lines reported here represent a new addition in the Drosophila toolkit to address the genetic basis of tissue specific antiviral immunity in this model insect.
